# A Standardized Extract of *Lentinula edodes* Cultured Mycelium Inhibits *Pseudomonas aeruginosa* Infectivity Mechanisms

**DOI:** 10.3389/fmicb.2022.814448

**Published:** 2022-03-16

**Authors:** Mireia Tena-Garitaonaindia, Diego Ceacero-Heras, María Del Mar Maldonado Montoro, Fermín Sánchez de Medina, Olga Martínez-Augustin, Abdelali Daddaoua

**Affiliations:** ^1^Department of Biochemistry and Molecular Biology II, Pharmacy School, University of Granada, Granada, Spain; ^2^Clinical Analysis Service, Hospital Campus de la Salud, Granada, Spain; ^3^Instituto de Investigación Biosanitaria (IBS), Granada, Spain; ^4^Department of Pharmacology, School of Pharmacy, University of Granada, Granada, Spain; ^5^Centro de Investigación Biomédica en Red de Enfermedades Hepáticas y Digestivas (CIBERehd), Madrid, Spain; ^6^Institute of Nutrition and Food Technology “José Mataix,” Center of Biomedical Research, University of Granada, Granada, Spain

**Keywords:** prebiotic, AHCC^®^, *Pseudomonas aeruginosa*, motility and biofilm, secretion system and adhesion, immune response, PCR real time (qPCR), internalization

## Abstract

The priority pathogen list of the World Health Organization classified *Pseudomonas aeruginosa* as the second top critical pathogen. Hence, the development of novel antibacterial strategies to tackle this bacterium is highly necessary. Herein we explore the potential antibacterial effect of a standardized extract of cultured mycelium of *Lentinula edodes* (AHCC^®^) on *P. aeruginosa*. AHCC^®^ was found to inhibit the growth rate and biofilm formation of strain PAO1. No change in swarming was observed, but AHCC^®^ hampered swimming and twitching motility. In accordance, a decreased expression of metabolism, growth, and biofilm formation genes was shown. AHCC^®^ also diminished the levels of exotoxin A and bacteria inside IEC18 cells and the secretion of IL-6, IL-10 and TNF by infected macrophages. This effect was related to a reduced phosphorylation of MAPKs and to bacteria internalization. Taken together, our data suggest that AHCC^®^ has a potential role to prevent *P. aeruginosa* infections and may lead to the development of new therapies.

## Introduction

The gram-negative *Pseudomonas aeruginosa* is a versatile and opportunistic nosocomial pathogen able to infect different animals and plants, and ubiquitously present due to its phenomenal capacity to adapt to different environments (Goldberg, 2010). *P. aeruginosa* is one of the major pathogens causing hospital-acquired infections, including ventilator associated pneumonia (Chastre and Fagon, 2002), catheter infections in immuno-compromised patients and respiratory infections in cystic fibrosis patients (CF) (Hassett et al., 2010) and severely burned people (Montie et al., 1982a,b). Furthermore, conditions like prolonged antibiotic treatment, metabolic syndrome and intestinal inflammatory diseases, that compromise the intestinal barrier function and cause dysbiosis, facilitate the establishment of *P. aeruginosa* in the intestinal ecosystem (Von Klitzing et al., 2017). This pathogen possesses a high level of intrinsic resistance to most antibiotics and, consequently, *P. aeruginosa* infections have become a worldwide health concern (Tacconelli and Magrini, 2017), the development of novel antibacterial strategies to tackle this pathogen being of critical importance. In fact, *P. aeruginosa* ranks second in the World Health Organization (WHO) priority pathogen list; a list of 12 species of bacteria highly resistant to antibiotics, elaborated to help prioritizing the research and development of new and effective antibiotic treatments.

*Pseudomonas aeruginosa* has developed a series of mechanisms to resist the effect of antibiotics and enhance virulence, including the synthesis of exotoxin A, a virulent factor (Iglewski and Kabat, 1975; Ortiz-Castro et al., 2014) that induces eukaryotic cell death (Hamood et al., 2004) and the formation of biofilms (Mah et al., 2003; Zhang and Mah, 2008; Taylor et al., 2014). Other virulence factors allow this pathogen to interact with epithelial cell monolayers and cross them. In this sense, its single polar flagellum facilitates near-surface swimming and attachment, and retractile type 4 pili power adhesion to and movement across biotic surfaces. Different secretion systems are then used to inject virulence factors into the cytoplasm of eukaryotic cells, allowing bacterial replication within macrophages or intestinal cells and the subsequent evasion from the immune system. In this context, six different classes of secretion systems [type I (TS1SS) to VI (TIVSS)] have been described so far (Durand et al., 2009; Bleves et al., 2010).

The different types differ in their molecular mechanisms and operate on different substrates. The type III secretion system (T3SS) of many bacterial pathogens injects a subset of bacterial “effector” proteins into host cells and in turn triggers a complete cytoskeletal reorganization, thereby preventing further uptake of bacteria. Further, ExsA, a member of the AraC/XylS family of transcriptional regulators (Hauser, 2009; Parsot, 2009), controls the transcription of T3SS genes.

In addition, another secretion system, termed type VI (T6SS) (Llosa et al., 2009), which shows similarities with the type III system (Cossart and Sansonetti, 2004; Merrell and Falkow, 2004), induces the expression of genes that encode the haemolysin-coregulated protein (Hcp) and the valine glycine repeat G (VgrG) family of proteins VgrG1, VgrG2 and VgrG3, that form part of the T6SS gene cluster (Suarez et al., 2008, 2010).

Different strategies are being developed to identify new drugs with direct and effective antibacterial activity that could be used as agents against *P. aeruginosa* (Siles et al., 2013; Rangel-Vega et al., 2015; Wilkinson and Pritchard, 2015; Torres et al., 2016), or as combinatorial drugs increasing the effectiveness of conventional antibacterials (Delattin et al., 2014; Van den Driessche et al., 2017). A significant number of natural compounds have been found to inhibit bacterial growth, although their mechanisms of action, in many cases, remain unclear (Johansson et al., 2008; Amer et al., 2010; Ortega-González et al., 2014; Rubio-Gómez et al., 2020). Among proposed foods or food components, prebiotics have been shown to perform an antagonistic role against gastrointestinal pathogenic bacteria (Oelschlaeger, 2010). Indirect mechanisms exerted by prebiotic on non-pathogenic bacteria or eukaryotic cells to inhibit bacterial growth have been described. These include the modulation of the host immunity or the induction of beneficial bacteria that compete for nutrients, produce antimicrobial peptides, or block the adhesion of pathogens to intestinal epithelial cells (Collado et al., 2007). Direct effects on specific pathogenic bacteria have been also studied. In this sense, we have described that inulin derived oligosaccharides modulates *P. aeruginosa* gene expression regulating biofilm formation and motility interfering with different signaling pathways (Ortega-González et al., 2014; Rubio-Gómez et al., 2020).

The standardized extract of cultured mycelium of *Lentinula edodes* (AHCC^®^) is a fermented extract that is available as a dietary supplement. AHCC^®^ contains a mixture of oligosaccharides (∼74% of dry weight, ∼20% being of the α-1,4-glucan type), amino acids, lipids, and minerals (Kidd, 2005). The acetylated forms of α-1,4-glucans are resistant to digestion, have low molecular masses (∼5 kDa) and are believed to constitute its main active ingredient (Matsui et al., 2002). In addition, AHCC also contains β-(1, 3)-glucans with β-(1, 6) branches, also resistant to digestion. In fact, we have previously shown that AHCC^®^ has intestinal anti-inflammatory and prebiotic effects in animal models of colitis, inhibiting the growth of pathogenic bacteria (Daddaoua et al., 2006, 2007). AHCC^®^ is well tolerated both as a human nutritional supplement and as a therapeutic agent (Ghoneum et al., 1995; Kidd, 2005). As such, it is used to reduce adverse effects observed in advanced cancer patients during chemotherapy and growing evidence indicates multiple roles of AHCC^®^ in cancer [including breast (Ito et *al*., 2014), ovary (Choi et al., 2018) and pancreas (Yanagimoto et al., 2016)], host protection during viral (Roman et al., 2013) or bacterial infections (Aviles et al., 2008), chronic diseases, diabetes (Ye et al., 2003) and cardiovascular pathologies (Corradetti et al., 2019).

Our research group has described that AHCC^®^ directly regulates the immune system acting on macrophage and intestinal epithelial cells (IEC18), an effect that involves toll like receptors and the adaptor molecule MyD88 and signaling pathways that include NF-κB/MAPK activation (Daddaoua et al., 2013). Based on these observations, and its prebiotic effects, here we have tested the hypothesis that AHCC^®^ could be useful in the regulation of the growth and virulence of *P. aeruginosa.* With this aim, we have characterized its direct effect on *P. aeruginosa* growth, mobility, biofilm formation, and gene expression of virulence factors. Moreover, intestinal epithelial cells (IEC18) cultures have also been used to assess the effect of AHCC^®^ on the capacity of *P. aeruginosa* to infect innate immune cells and produce an inflammatory response.

## Materials and Methods

### Materials

AHCC^®^ (Amino Up Co., Ltd., Sapporo, Japan) is a standardized extract of cultured *Lentinula edodes* mycelia. The solutions were made at 50 g/L in modified LB medium and, in the case of the eukaryotic cells, in Dulbecco’s Modified Eagle’s Medium (DMEM Sigma^®^) containing fetal bovine serum (FBS, 10%), 2 mM L-glutamine, and 2.5 mg/mL amphotericin B. Solutions were filtered using 0.22 μm cut-off filters.

### Bacterial Strains Used in This Study

*Pseudomonas aeruginosa* PAO1 was grown in LB, modified M9 minimal medium (Abril et al., 1989) or complete Brain Heart Infusion (BHI) medium. When required, antibiotics were added to the culture medium to reach a final concentration of 50 μg/mL ampicillin.

### Cell Lines

To isolate macrophages, female Wistar rats were sacrificed and the spleen was extracted aseptically. Cell suspensions were obtained by disrupting the spleen between dissecting forceps in physiological in medium. After centrifugation (1,000 × *g*/5 min), cells were cleared of erythrocytes by resuspension in hypotonic lysis buffer (15 mM NH4Cl, 10 mM KHCO3, 0.1 mM Na2EDTA, pH 7.3) for 30 min on ice. Mononuclear cells were washed and resuspended in MACs buffer (PBS containing 0.5% (w/v) BSA, 2 mM and EDTA, pH 7.2). To obtain a monocellular suspension, cells were passed through 70 μm nylon mesh prior to magnetic labeling and subsequently isolated by negative selection. To remove lymphocytes, CD161.1-biotin (1:200), CD45RA-PE (1:200) and CD3 (1:150) (Biosciences), were added and incubated at 4°C for 30 min. Cells were washed and sedimented by centrifugation at 1,000 × *g* for 5 min. After resuspension in MACs buffer, 25 μL of each Microbeads and anti-PE-microbeads (Miltenyi Biotec), were added and the resulting suspension incubated at 4°C during 30 min. Cells were washed, centrifuged and dissolved in DMEM medium (Dulbecco’s Modified Eagle Medium). CD161.1 +, CD45RA + and CD3 + cells were discarded using an LD column (Miltenyi Biotec). Macrophages in the flow-through were centrifuged at 1,000 × *g* for 5 min and resuspended in Dulbecco’s Modified Eagle Medium (DMEM) supplemented with 10% FBS (Sigma), 2.5 mg/L amphotericin B and 2 mM L-glutamine.

Intestinal epithelial IEC18 cells (passages 20–32) were obtained from the Cell Culture Service of the University of Granada and cultured in DMEM containing 10% FBS, 2 mM l-glutamine, 100 mg/L streptomycin, 100,000 U/L penicillin and 2.5 mg/L amphotericin B, in standard conditions.

### Effects of AHCC^®^ on *P. aeruginosa* Growth

Individual colonies of *P. aeruginosa* PAO1 were picked from the surface of freshly grown LB plates and grown overnight in M9 minimum medium supplemented with 5 mM of citrate or in complete Brain Heart Infusion (BHI) medium at 37°C. The overnight culture was diluted to an OD_660nm_ of 0.05 or 0.6 with M9 minimum medium and of 0.05 with BHI medium. Ninety-six well flat-bottomed polystyrene microtiter plates were filled with 180 μL of bacterial suspensions. AHCC^®^ was added to bacteria grown in M9 from OD = 0.05 and BHI media, to reach final concentrations of 5, 15 and 30 mg/mL, while 5 mg/mL of AHCC was added to bacteria grown in M9 medium at OD = 0.6. Plates were incubated at 37°C under continuous agitation in a Bioscreen C MBR analyser type system FP-1100-C (OY Growth Curves Ab Ltd., Raisio, Finland). The turbidity was measured at 580 nm, using a wideband filter at 420–660 nm every 60 min, over a 24 h period for M9 medium starting at OD = 0.05. A 660 nm filter and a follow up period of 8 h was used when bacteria were cultured in BHI or M9 medium starting at OD = 0.6. Both procedures are equivalent for measuring bacterial growth in suspension and one or the other were used for purely practical reasons.

### Semiquantitative Determination of Biofilm Formation

The inhibitory effect of AHCC^®^ on *P. aeruginosa* biofilm formation was measured as described (Christensen et al., 1985). The growth was initiated by inoculating 500 μL of M9 medium with 5 μL of suspended bacteria from an overnight culture, to reach a DO_660nm_ of 0.05. Bacteria were cultured in individual wells of a 24-well microtiter plate at 37°C for different times in the absence and in the presence of different concentrations (up to 20 mg/mL) of AHCC^®^.

Biofilm formation was quantified using the crystal violet (CV) method (Fredheim et al., 2009). The structure of biofilm was observed under contrast-phase microscopy using a Zeiss Axioscope fluorescence microscope coupled to a Nikon DSS-Mc CCD camera and a 100-fold magnifier. Data reported are means from two independent experiments each conducted in quadruplet repeats.

### Motility Assays

Assays were carried out to determine the effect of AHCC^®^ (5, 15 and 30 mg/mL) on swimming, twitching and swarming motility. In all assays these compounds were added at identical concentrations to the plates containing AHCC. For swimming assays bacteria were placed with the help of a sterile tooth-pick at the center of plates containing a 5 mm layer of LB medium with 0.3% (w/v) Bacto agar, 0.2% casamino acids (w/v) and 30 mM glucose. Plates were incubated at 37°C for 24 h and the radial diffusion of bacteria, due to swimming, was inspected. To monitor twitching motility bacteria were placed with a toothpick into a 2 mm thick layer containing 1.5% (w/v) Bacto agar, 0.2% (w/v) casamino acids and 30 mM glucose. After incubation for 24 h at 37°C, the expansion of bacteria on the plate was observed. For swarming assays 5 μL of an overnight culture of bacteria in minimum medium M9 were diluted to OD 0.6 and placed into the center of swarm plates (composed of 0.5% (w/v) Bacto agar supplement with 0.2% (w/v) casamino acids and 30 mM glucose). Plates were incubated at 37°C for 24 h, followed by an inspection of the surface movement of the bacteria. All the motility assays were performed in triplicates.

### Measurement of AHCC^®^-Induced Lactate Dehydrogenase Release in Macrophages and IEC18 Epithelial Cells

Cellular toxicity was quantified by the measurement of lactate dehydrogenase (LDH) release using non-radioactive Pierce LDH Cytotoxicity Assay Kit (Thermo Scientific), following the manufacturer’s instructions. IEC18 cells and rat macrophage suspensions (10^6^–10^8^ of cells/mL DMEM medium) were incubated with different concentrations of AHCC^®^ at 37°C for 8 h and 24 h, respectively. LDH release was measured in the culture supernatant by colorimetric detection at 490 and 680 nm.

### Measurement of the Effect of AHCC^®^ on Cytokine Secretion From Macrophage and IEC18 Cells Following Infection by *P. aeruginosa* PAO1

For the determination of cytokines macrophage suspensions (10^6^–10^8^ of cells/mL DMEM medium) and IEC18 cells were cultured with *P. aeruginosa* and incubated with non-toxic concentrations at 2 and 5 mg/mL of AHCC^®^ for 8 h. Following centrifugation of macrophage suspensions at 4°C and 10,000 × *g* for 5 min, the resulting supernatants were frozen at −80°C. Supernatants from IEC18 cells were also collected, centrifuged and frozen. Aliquots were thawed and cytokine levels determined using ELISA-based kits (BD Biosciences, Erembodegem, Belgium) following the protocol provided by the manufacturer. In addition, macrophage cells were used for the quantification of IkB and MAP kinase by western blot as described below.

### Western Blot Assays

For the detection of p-ERK (the phosphorylated form of Extracellular Regulated Kinase), p-P38 (phosphorylated form of P38 mitogen-activated protein kinases), p-JNK (phosphorylated Jun N-terminal kinase), p-IkB (phosphorylaated form of IkappaB kinase) cells were homogenized in lysis buffer (PBS containing 0.1% (w/v) SDS, 0.1% (w/v) sodium deoxycholate, 1% (v/v) Triton X-100) with protease inhibitor cocktail (Sigma) 1:100 (v/v). Subsequently, homogenates were sonicated and centrifuged 7,000 × *g* for 5 min at 4°C. Protein concentrations were determined using the bicinchoninic acid assay (Ortega-González et al., 2014). Samples were boiled in 5 × LaemmLi buffer (220 mM Tris, 312 μM SDS, 50% (v/v) glycerol, 1% (v/v) 2-mercaptoethanol, 22.5 mM EDTA, pH 6.8, containing traces of bromophenol blue) for 5 min, separated by SDS-PAGE, electroblotted to PVDF membranes (Millipore, Madrid, Spain), and exposed to the primary antibodies against ERK, p-ERK (both from Sigma), p-P38, p-JNK and phosphorylated p-IkB, respectively (all from Cell signaling, Danvers, MA, United States). Prior to exposure to the secondary IgG Peroxidase antibody (provided by Sigma, mouse for ERK and p-ERK, rabbit in the others) the bands were visualized by enhanced chemiluminescence (PerkinElmer, United States) and quantified with the NIH software (Scion Image).

### Determination of Exotoxin A Concentration in IEC18 Cells

The cells were cultured in 6 well-plates. At confluence, cells were infected with *P. aeruginosa* at a ratio of 5 bacterial cells per eukaryotic cell in the absence and in the presence of non-toxic concentrations (2 and 5 mg/mL) of AHCC^®^. Plates containing cells were washed 3 times with PBS and incubated with a gentamicin solution (100 mg/mL) for 1 h to eliminate bacteria. Subsequently, plates were washed 3 times with PBS prior to cell collection using RIPA buffer containing a protease and phosphatase inhibitor cocktail (Sigma). Proteins were extracted and Western blots were carried out as outlined above using the Exotoxin A antibody (Sigma) at a 1:2,000 dilution. Following overnight incubation and washing with the primary antibody the membrane was incubated with the secondary IgG Peroxidase anti-rabbit antibody (Sigma) at a 1:10.000 dilution for 2 h. The bands were detected by enhanced chemiluminescence (PerkinElmer, Waltham, MA, United States) and quantified with NIH software (Scion Image).

### Analysis of Gene Expression by Quantitative Real Time PCR

The expression of the genes was studied using quantitative real time PCR. Total RNA of *P. aeruginosa* culture, in the presence and in the absence of 2–5 mg/mL of AHCC^®^ for 8 h, was obtained by the TRI reagent^®^/BCP method (Invitrogen). AHCC^®^ was added to *P. aeruginosa* grown to OD = 0.6. 1 μg of RNA was retrotranscribed following the protocol recommended by the manufacturer (iScript, BioRad, Alcobendas, Spain) and specific RNA sequences were amplified with MX3005P real time PCR device (Stratagene) using the set of primers pairs indicated in Supplementary Table 1. The genes of interest were amplified by PCR (61°C as annealing temperature, 40 cycles), through Go Taq^®^qPCR Master Mix (PromegaMadison, United States), using 2 ml cDNA as template. The cycle threshold values were normalized to that of the reference transcript, 16S RNA, and a fold change compared to the control group was calculated.

### Bacterial Internalization by Cultured IEC18 Cells

The internalization of viable bacteria in IEC18 cells was tested using an overnight culture of *P. aeruginosa* PAO1 strain. Subsequently, the bacteria were washed twice and diluted in the DMEM medium. For infection, a ratio of 10^6^–10^8^ (Bacteria/IEC18 cells) was maintained. Previously, IEC18 cells were treated with cytochalasin D (CytoD) at a non-toxic concentration of 2 μM and were synchronously infected with bacteria/cell for 2 h in the presence and in the absence of AHCC^®^ at 2 and 5 mg/mL of final non-toxic concentration. To remove the non-phagocytosed bacteria the eukaryotic cells were washed three times in ice-cold PBS and further harvested and lysed in a lysis buffer [PBS containing 0.1% (w/v) SDS, 0.1% (w/v) sodium deoxycholate, 1% (v/v) Triton X-100] with protease inhibitor cocktail (Sigma) 1:100 (v/v). The internalization of *P. aeruginosa* was analyzed by plating a series of dilutions 10^–5^ and 10^–10^ on LB agar and incubating at 37°C overnight. The number of bacteria phagocytosed into IEC18 epithelial cells was determined by counting the growth colony forming units (CFU).

### Statistical Analysis

All results are expressed as means with the corresponding standard deviations. Differences among means were analyzed for statistical significance by a one-way ANOVA analysis and *a posteriori* least significance test. All analyses were carried out with the GraphPad Prism 7.0 program (Jandel Corporation, San Rafael, CA, United States). Concentration-response curves were fitted to a logarithmic curve when possible with Origin 7.0 (OriginLab Corporation, Northampton, MA, United States). Differences were considered significant at *P* < 0.05.

## Results

### Effect of AHCC^®^ on the Growth of *P. aeruginosa* PAO1

**FIGURE 1:**
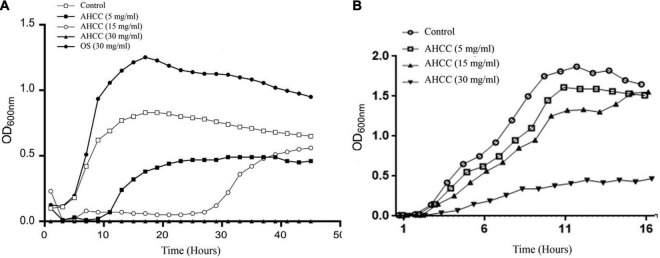
Effect of AHCC^®^ on the growth of *P. aeruginosa*. The growth curves in minimal medium M9 **(A)** and complete medium BHI **(B)** in the absence and presence of AHCC^®^ at 5, 15 and 30 mg/mL are shown. As a control for the use of oligosaccharides as a carbon source when bacteria were grown in minimal medium M9 20 mg/mL of goat milk oligosaccharides (OS) was used. Growth curves were recorded at 37°C for 24 h. Representative data from one of three independent experiments with similar results are shown.

AHCC^®^ is a standardized extract of cultured *Lentinula edodes* mycelia which contains a mixture of oligosaccharides, including digestion resistant glucans. To explore the effect of AHCC^®^ on *P. aeruginosa*, growth curves were obtained in M9 medium (Figure 1A) and in BHI medium (Figure 1B). Two different strategies were followed using M9 medium: inoculating bacteria to the medium and following the growth for 24 h in the presence of AHCC^®^ or growing the bacteria to exponential phase (OD = 0.6) before adding AHCC^®^. When bacteria were added to M9 medium (OD = 0.05) in the presence of 5 and 15 mg/mL of AHCC^®^ a pronounced reduction in bacterial growth rate and yield was noted, suggesting antibacterial activity. However, in the presence of 30 mg/mL of AHCC^®^ a complete growth inhibition was observed, probably due to the toxic effect of AHCC^®^ at this high concentration (Figure 1A). Additionally, a growth curve was generated in the presence of 30 mg/mL of control oligosaccharides (OS) as a carbon and energy source, which showed that OS promoted bacterial growth (Figure 1A). The experiments carried out with BHI medium, in which AHCC^®^ was added at OD = 0.05, indicate that the lower concentration of AHCC^®^ (5 mg/mL) had no substantial effect on P. *aeruginosa*, and only when the bacteria were cultured with AHCC^®^ 30 mg/mL a significant growth inhibition was observed (Figure 1B). Finally, when AHCC^®^ was added to bacteria grown to OD = 0.6 no substantial effect was observed (Figure 1B). Therefore, our results indicate that AHCC^®^ could be toxic at high concentrations. In addition, in a minimum medium (M9) a low concentration of AHCC^®^ (5 mg/mL) may inhibit growth when bacteria are diluted, but this effect was essentially absent in a complete medium or with concentrated bacteria (OD = 0.6) in minimal medium, indicating that AHCC at the concentrations used in other experiments in this study is not toxic to *P. aeruginosa*.

### Biofilm Formation and Quantification in the Presence of AHCC^®^

**FIGURE 2:**
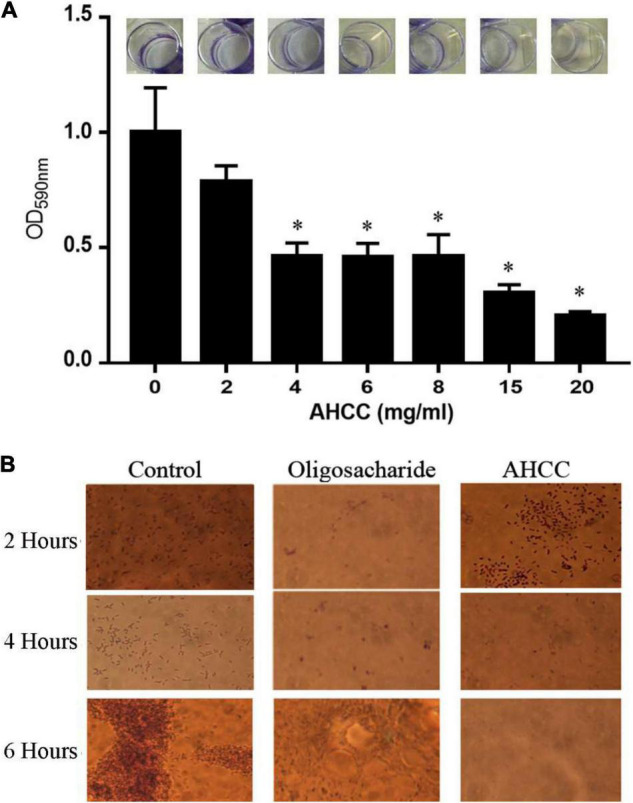
Formation of *P. aeruginosa* biofilm. **(A)** Biofilm formation in the absence and in the presence of different concentrations of AHCC^®^ in 24-well plates. Biofilm formation was monitored in M9 minimal medium supplemented with 0.2% (w/v) glucose and casamino acids and quantified after 6 h. Then, the relative amount of biofilm formation in the experiments was represented against AHCC^®^ concentration. Data are the average of three independent assays. **(B)** Microscopic inspection of biofilm formation in the absence and in the presence of 10 mg/mL of AHCC^®^ at 2, 4 and 6 h.

Biofilm formation is a mechanism used by *P. aeruginosa* for surface colonization that leads to a significant resistance against antibiotics. To explore the inhibitory effects of AHCC^®^ on *P. aeruginosa* biofilm formation, the growth was initiated by inoculating 500 μL of LB medium with 5 μL of suspended bacteria, from an overnight culture to reach DO_660nm_ of 0.05, in individual wells of a 24-well microtiter plate and then incubated at 37°C for different times (Figure 2 and Supplementary Figure 1). The biofilm formation was quantified after 4–8 h using the crystal violet (CV) method as described in material and methods (Figure 2 and Supplementary Figure 1). The Figure 2A shows that biofilm formation was clearly inhibited (50–70 fold) in a concentration-dependent manner by AHCC^®^ (2–20 mg/mL) for 4 h. Subsequently, this inhibitory effect was confirmed by the inspection of biofilm formation under the microscope (Figure 2B). These results suggest that AHCC^®^ is potentially useful in preventing biofilm formation either directly or indirectly by inhibiting growth.

### AHCC^®^ Effect on Bacterial Motility

**FIGURE 3:**
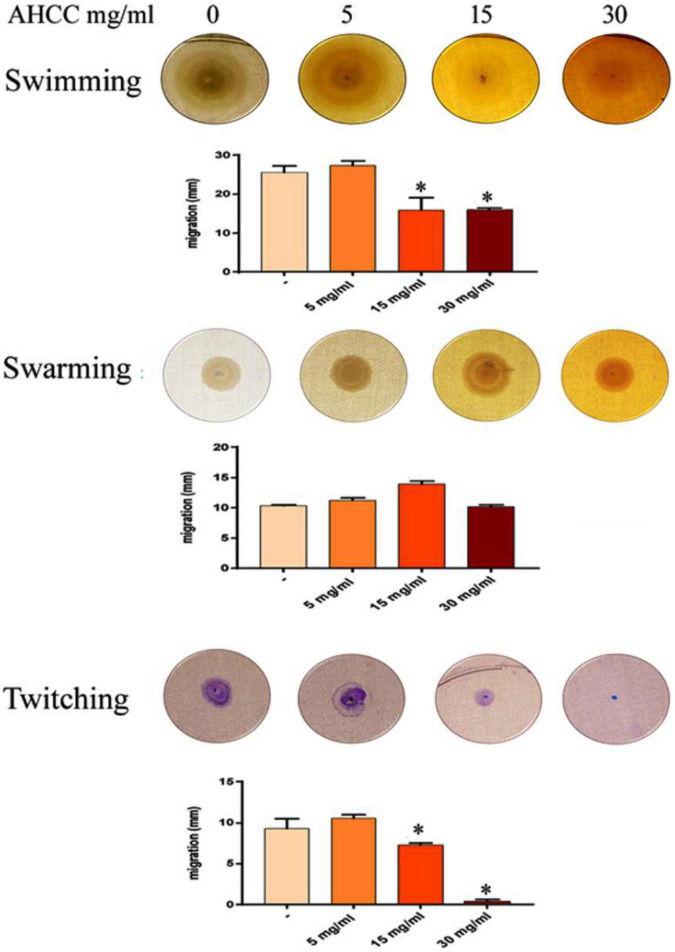
Effects of AHCC^®^ on the motility of *P. aeruginosa.* Motility assays were carried out as described in Materials and Methods. AHCC^®^ at 5, 15 and 30 mg/mL was present in the agar plates and in the bacterial suspension. *p < 0.05 vs. control (ANOVA followed by least significance tests).

*Pseudomonas aeruginosa* exhibits three different types of motility, namely swimming, swarming and twitching, which play a key role in the colonization of surfaces by bacteria and the subsequent formation of biofilms (Mattick, 2002; Klausen et al., 2003). Initial experiments investigated the effects that AHCC^®^ extract (5, 15 and 30 mg/mL) had on the levels of *P. aeruginosa* strain PAO1 swimming motility, surface-associated swarming and twitching motilities. Under control conditions (AHCC^®^ free), this bacterium was able to undertake all three types of motility (Figure 3). Nevertheless, while AHCC^®^ caused no change in swarming motility, it was found that it significantly inhibited (at least 50% of controls) the swimming and twitching motility at 15 and 30 mg/mL (*p* < 0.05). Interestingly, AHCC^®^ completely blocked twitching motility when plates were incubated in the presence of 30 mg/mL of AHCC^®^ concentration at 24 h time point (Figure 3). The effect on swimming and twitching motility may be due to direct modulation by AHCC^®^, to a growth inhibitory effect, or to a combination of both.

### AHCC^®^ Reduces Transcript Levels for Genes That Are Related to Bacterial Pathogenicity

Quantitative real time PCR (RT-qPCR) data analysis shows that AHCC^®^ reduces the transcript levels for PA0807 (*ampD*) and PA0908 (*alpB*), encoding proteins involved in responses to antibiotics, suggesting that AHCC^®^ modulates the sensitivity to antibiotics (Amber and Nancy, 2008; Shah et al., 2008; Alvarez-Ortega et al., 2010). Moreover, the results shown in Table 1 evidence reduced transcript levels of the *dctA* gene (PA1183), associated with the normal growth of *P. aeruginosa* (Valentini et al., 2011), and of the *icmP* gene (PA4370), encoding a metalloproteinase outer membrane protein which has been shown to degrade plasminogen activator (Christian et al., 2015) and to play a key role in *P. aeruginosa* pathogenicity. However, the reduced transcript level of the gene (PA3866) encoding the pyocin S4 suggests that AHCC^®^ acts as a signal molecule that modulates bacterial virulence through distinct signaling pathways, including partial growth inhibition.

**TABLE 1:** Quantitative real time PCR experiments to quantify the effect of AHCC§on the transcript levels of genes which are related to bacterial pathogenicity in *P. aeruginosa* infected IEC18 cells.

Gene ID	Gene	Protein	Relative expression levels	*P* value
PA0807	*ampD*	*N*-acetylmuramoyl-L-alanine amidase	−3.2	0.1798
PA0908	*alpB*	Outer membrane protein AlpB	−4.12	0.175
PA1183	*dctA*	C4-dicarboxylate transport protein	−6.2	0.0061
PA3866	*Pyocin S4*	soluble (S-type) pyocins	−13.22	0.0373
PA4370	*icmP*	Insulin-cleaving metalloproteinase outer membrane	−5.64	0.0163

*Experiments were conducted using IEC18 cells infected with P. aeruginosa cells (ratio 1/5) for 4 h in either the absence or the presence of 5 mg/mL AHCC prior to carrying out the PCR-real time.*

*The cycle threshold values were normalized to that of the reference transcript, 16S RNA, and data of relative expression levels were normalized to the control lacking AHCC.*

### AHCC^®^ Is Not Toxic to IEC18 Cells or Rat Macrophages

**FIGURE 4:**
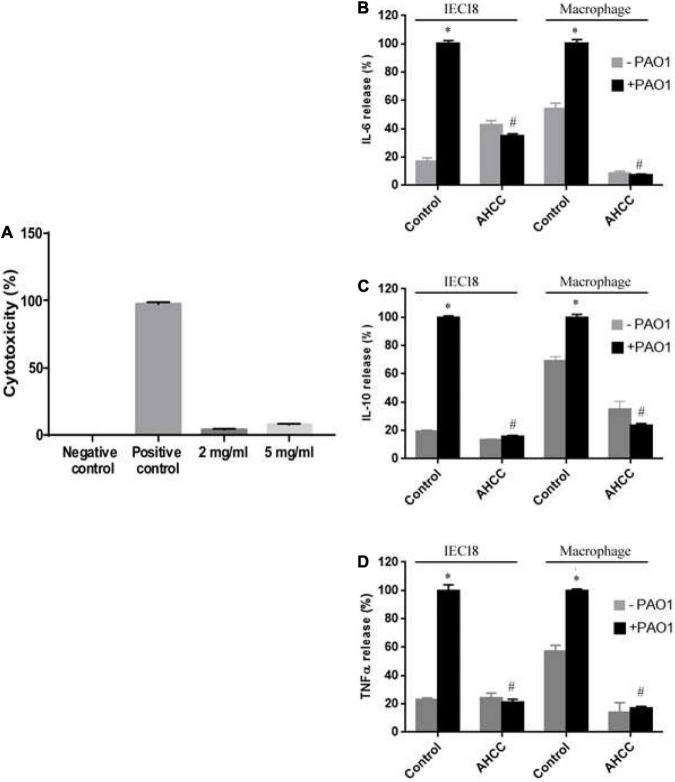
Toxicity and inflammatory response of AHCC^®^ on macrophages and IEC 18 cells. Lactate dehydrogenase (LDH) as a marker of cytotoxicity was measured in the supernatants of IEC18 cells **(A)** exposed to AHCC^®^ at 2 or 5 mg/mL. **(B–D)** Cytokine levels in the supernatant of IEC18 or macrophages. Cells were incubated with *P. aeruginosa* PAO1 (ratio 1/5) for 8 h in either the absence or the presence of 5 mg/mL AHCC^®^ prior to the determination of IL-6 **(B)**, IL-10 **(C)** and TNF secretion **(D)**. Values are means ± s.e.m., *n* = 6–8; *p < 0.05 vs. cells without bacteria and ^#^*p* < 0.05 vs. WT in the absence of AHCC^®^ (ANOVA followed by least significance tests).

To assess the possible toxic effect of AHCC^®^ in IEC18 cells and macrophages, we quantified the level of lactate dehydrogenase (LDH) activity in the culture medium, a marker of cytotoxicity. As described by Ortega-González et al. (2014), Figure 4A shows that AHCC^®^ does not affect cell viability as demonstrated by the low lactate dehydrogenase activity in the medium compared to the LDH released in the presence of the positive control (Figure 4A). In parallel, AHCC was shown not to alter LDH activity in the medium of rat macrophages cultured for up to 24 h after exposure (Supplementary Figure 2). Therefore, the 5 mg/mL concentration was selected for further experiments.

### AHCC^®^ Reduces Cytokine Secretion by Rat Macrophages and IEC18 Cells Infected With *P. aeruginosa*

The IL-6, IL-10 and TNF cytokines are produced by activated macrophage cells as a positive response of inflammatory reactions in the process of bacterial infection. These cytokines are also produced, although in much lower concentrations, by IEC18 cells. To examine the specific effect of AHCC^®^ on cytokine production by rat macrophages and IEC18 cells infected with *P. aeruginosa* PAO1 strain, the secretion of cytokines (IL-6, IL-10 and TNF-α) at 8 h was investigated in the presence and in the absence of AHCC^®^ at 5 mg/mL (Figure 4) using enzyme-linked immunosorbent assay (ELISA) kits (Invitrogen, Thermo Fisher Scientific). The data in Figures 4B,D, respectively, indicate that the presence of AHCC^®^ reduced significantly the pro-inflammatory cytokine (IL-6 and TNF-α) and the anti-inflammatory cytokine (IL-10) (Figure 4C) compared with the cell infected by bacteria and without AHCC^®^, suggesting an anti-inflammatory effect of AHCC^®^ in both cell types.

### AHCC^®^ Modulated Signal Transduction Pathways in Macrophage Cells Infected With *P. aeruginosa*

The mitogen-activated protein kinase (MAPK) and the NFkB signaling pathways are implicated in the production of TNF-α and IL-6 in macrophage cells (Lee et al., 2010; Yang et al., 2007). To study the modulation exerted by AHCC^®^ on the inflammatory response. We have therefore studied the importance of NF-kB and MAPK signaling molecules in macrophage cell infected by *P. aeruginosa* during AHCC^®^ treatment through a western blot analysis.

**FIGURE 5:**
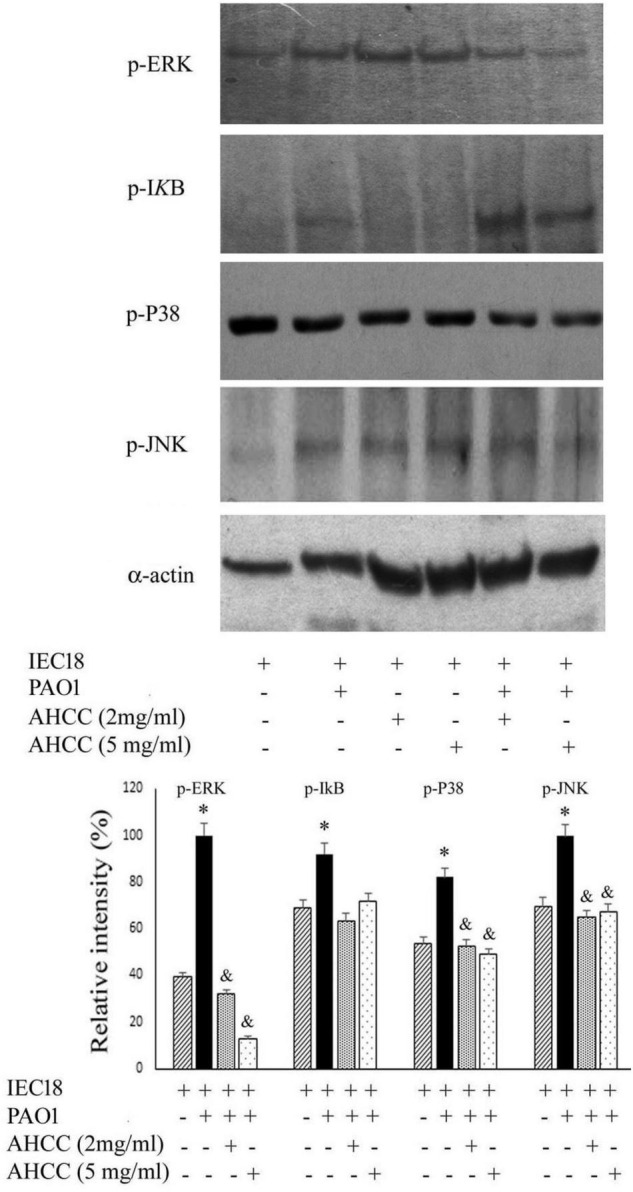
Inflammatory transduction pathways are modulated by AHCC^®^. Macrophage cells were cultured in the presence of *P. aeruginosa* in the absence or presence of 2 and 5 mg/mL AHCC^®^. After 8 h growth western blots were performed using the cell extracts and the corresponding antibody against specifics proteins. The following protein were detected: p-ERK (the phosphorylated form of Extracellular Regulated Kinase), p-P38 (Activated and phosphorylated form of P38 mitogen-activated protein kinases), p-JNK (Jun N-terminal kinases), p-IkB (phosphorylated form). As control, actin was quantified in all samples using α-actin antibody. Shown are duplicate sample in the absence of added effectors and triplicate samples in the presence of AHCC^®^. *p < 0.05 vs cells without bacteria. (ANOVA followed by least significance tests).

As shown in Figure 5, western blot analysis demonstrated that, while the NFκB pathway was not widely affected by the presence of the AHCC^®^ at 2 and 5 mg/mL, a significant decrease was observed in the level of p-P38, pJNK and, most prominently, in p-ERK phosphorylation (Figure 5), pointing to a modulation of the MAPK canonical pathway resulting in a significant reduction of IL-6, IL-10 and TNF-α secretion (Figure 4).

### Secretion Systems and Exotoxin A of *Pseudomonas aeruginosa* Are Modulated by AHCC^®^

**FIGURE 6:**
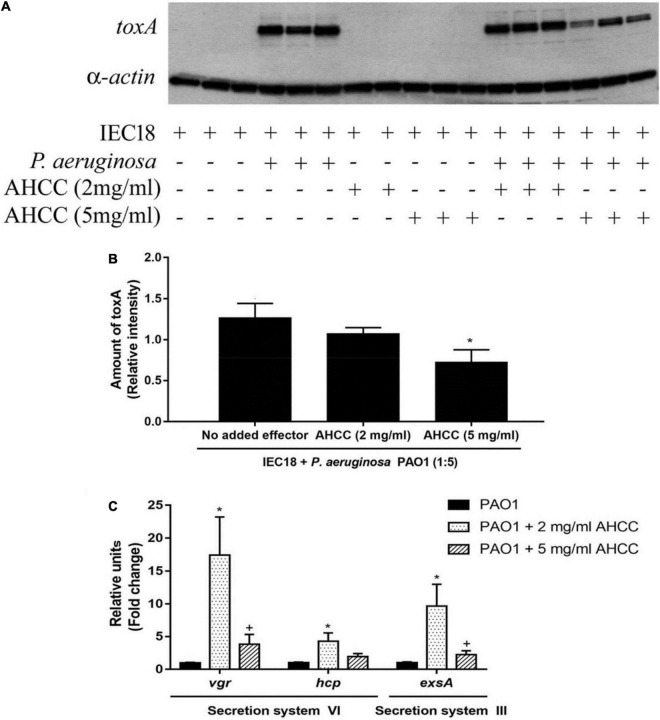
The gene expression of *P. aeruginosa* secretion systems and *toxA* are modulated by AHCC^®^. **(A)** Western blot determination of the cellular concentration of exotoxin A in IEC18 cells following co-culture with *P. aeruginosa* in the presence and absence of AHCC^®^ at 2 and 5 mg/mL of concentration. **(B)** Densitometric analysis of above data. Exotoxin A densities were corrected with those obtained for α-actin. **(C)** Quantitative real time PCR studies of *P. aeruginosa* of cultures grown in the presence and absence of AHCC^®^ at 2 and 5 mg/mL of concentration. The expression of genes encoding proteins of secretion systems III and VI are shown. Values are means ± S.E.M., *n* = 6–9; *p < 0.05 vs. without AHCC^®^ (ANOVA followed by least significance tests).

Many known natural products exert inhibitory effect on the secretion system which used by pathogenic bacteria to infect host cells (Ortega-González et al., 2014). To analyze the role of AHCC^®^ on *P. aeruginosa* virulence, internalized exotoxin A is quantified into IEC18 cells infected by bacteria. Anti-exotoxin A western blot analysis revealed that the addition of AHCC^®^ at 2 and 5 mg/mL to eukaryotic cells reduces significantly the levels of exotoxin A injected into the cell (Figures 6A,B). The data suggest that AHCC^®^ inhibits the mechanism of the type III and VI secretion system that allows exotoxin A to be injected into the eukaryotic cell. Nevertheless, the analysis, using real time qPCR, of expression genes encoding key proteins necessary for the function of secretion systems (i.e., ExsA/VgrG and Hcp for T3SS and T6SS, respectively), shows upregulation (Figure 6C). Thus, a mechanistic link for AHCC^®^-mediated reduction in intracellular exotoxin A concentration cannot be established at this level.

### Effect of AHCC^®^ on Inhibition of Actin Cytoskeletal Dynamics in IEC18 Cell on *P. aeruginosa* Internalization

The ability of pathogenic bacteria to interact with eukaryotic cells depends on the interaction of virulence factors such as adhesins and secretion systems (Gerlach and Hensel, 2007). To analyze whether the entry of the *P. aeruginosa* depends on the dynamics of the actin filaments, we studied bacterial internalization in the presence of actin depolymerizing fungal toxin cytochalasin D (Cyto D) to prevent bacterial internalization. This compound binds to the positive end of the F filament and prevents the addition of G actin monomers, inhibiting polymerization (Sampath and Pollard, 1991). The standard technique for quantifying internalized bacteria in these assays is still plating a series of dilutions on solid agar and counting the growth colony forming units (CFU).

**FIGURE 7:**
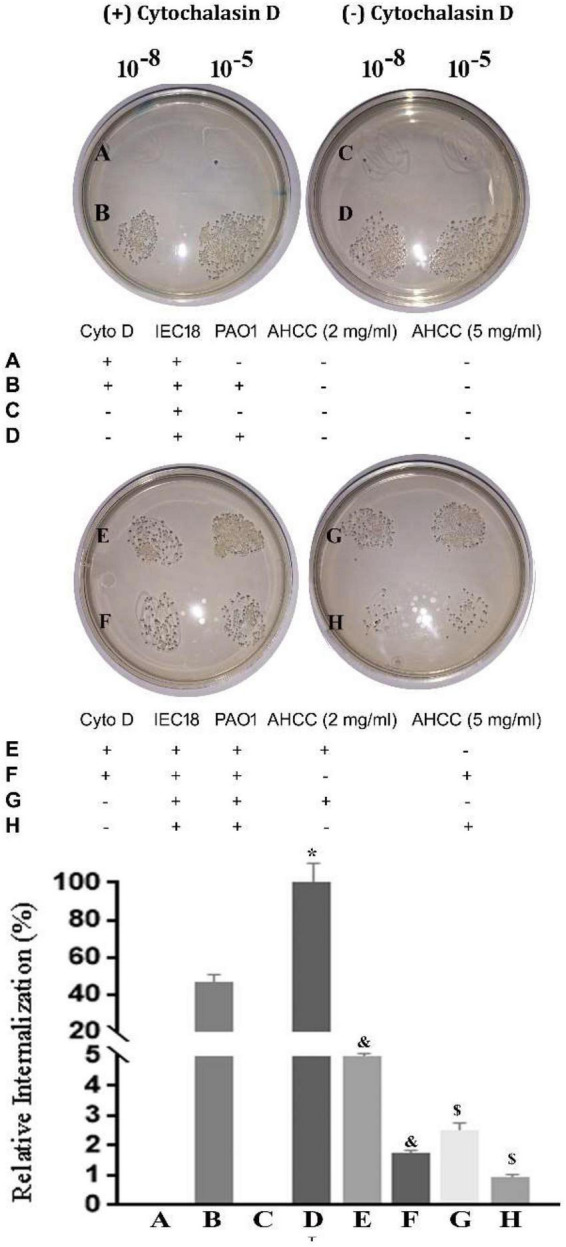
Effect of AHCC^®^ on internalization by IEC 18 *in vitro.* In the presence and in the absence of 8 μM Cyto D, the cells were synchronously infected with 10 CFU/cell for 60 min. Bacterial internalization was analyzed by automatically quantifying the growth colony forming units (CFU) (The experiment are performed in triplicated; mean ± S.E.M). The graph shows the mean percentage of bacterial internalization with respect to the sample control in the presence or absence of 20 mg/mL AHCC^®^. *p < 0.05 vs. without Cyto D and ^&^*p* < 0.05 vs. without AHCC^®^ (ANOVA followed by least significance tests).

As shown in Figure 7, bacterial internalization was around 55% lower in the presence of the pharmacological inhibitor Cyto D. Notably, addition of AHCC^®^ at either 2 or 5 mg/mL resulted in subtotal inhibition of bacterial internalization, suggesting that mechanisms unrelated to modulation of the cytoskeleton are involved in its effect. In fact, Cyto D had little effect compared with AHCC alone, pointing at a high impact of such mechanisms (Figure 7).

## Discussion

Development of new strategies to treat bacterial infections has become a health target, since the therapeutic arsenal is limited, and bacteria are developing multidrug resistances. *P. aeruginosa* presents limited susceptibility to antimicrobial agents and may become multidrug resistant. *P. aeruginosa* infections are a leading cause of nosocomial infections, responsible for 10% of all hospital-acquired infections, which are often severe and even life threatening (Aloush et al., 2006). These facts make of critical importance, according to the WHO, finding new strategies to prevent and treat *P. aeruginosa* infections.

Food and food components may be a good source in the search for alternatives to conventional antibiotics, due to their variety of components with low toxicity. Among them, prebiotics, including standardized extract of cultured *Lentinula edodes* mycelia (AHCC^®^), are known to regulate intestinal microbiome, through the regulation of the host immune system and the growth of beneficial bacteria. Effects on pathogens have also been described (Collado et al., 2007; Aviles et al., 2008), including *P. aeruginosa* (Ortega-González et al., 2014; Rubio-Gómez et al., 2020). Here, we have evaluated AHCC^®^ for the prevention of *P. aeruginosa* infection. The first approach has been to determine the effect of AHCC^®^ on bacterial growth, observing a concentration dependent longer lag phase and overall growth inhibition in minimal M9 medium (Figure 1). Accordingly, the analysis of gene expression indicated a reduction in the expression of the gene *dctA* (PA1183), which is associated with normal growth of *P. aeruginosa* (Valentini et al., 2011). Our data indicate that AHCC^®^ is not a good source of carbohydrates for *P. aeruginosa*, since these effects on the growth curve were observed when grown in M9 medium but not in BHI medium. In addition, we observed that once the log phase was reached (OD = 0.6) the addition of AHCC^®^ had little effect, possibly indicating that it has little or no direct effect on cell division.

On the other hand, AHCC^®^ affected several key aspects of the virulence of *P. aeruginosa*, including its motility and the subsequent formation of biofilms. *P. aeruginosa* utilizes flagellum-mediated swimming motility to approach a surface, where it attaches and further spreads *via* the surface-associated motilities swarming and twitching, mediated by multiple flagella and type-IV pili, respectively (Figure 3). AHCC^®^ reduced swimming motility and completely blocked twitching movements, while no changes in swarming were observed in our experimental conditions, indicating a differential inhibitory effect of AHCC^®^ on motility mechanisms. These may be largely related to reduced growth in the presence of AHCC^®^. Biofilms consist of bacteria encased within an extracellular matrix of proteins, polysaccharides, and small molecules. Biofilm formation provides increased protection of bacteria from antibiotics and host defenses (Mattick, 2002) and reduced biofilm formation has been related to the inhibition of movement capacity as swimming and twitching. Consistently with the reduced movement, a significant reduction in biofilm formation induced by AHCC^®^ was found for the first 4 h (Figure 2 and Supplementary Figure 1). The observed effects of AHCC^®^ increasing the lag phase of *P. aeruginosa*, matching the attaching phase and beginning of biofilm formation, may at least partly account for these effects.

Direct effects of AHCC^®^ on *P. aeruginosa* virulence were further characterized studying the expression of genes (PA0807, PA0908, PA4370 and PA3866) involved in antibiotic resistance and pathogenicity. PA0807 (*ampD*) and PA0908 (*alpB*) encode proteins whose increased expression is related antibiotic resistance (Amber and Nancy, 2008; Shah and Swiatlo, 2008; Alvarez-Ortega et al., 2010). The *IcmP* gene (PA4370) product is a metalloproteinase outer membrane protein which has been shown to degrade plasminogen activator (Christian et al., 2015) and to play a key role in *P. aeruginosa* pathogenicity. On the other hand, S-type pyocins are bacteriocins produced by *P. aeruginosa* isolates to antagonize or kill other strains of the same species, in other to avoid competition (Dingemans et al., 2016). These pyocins are two component systems consisting of a large and a small component, the large component being the killing subunit. Pyocin S4, encoded by the gene PA3866, is a large component with a C-terminal domain consistent with tRNase activity (Ameer et al., 2012).

The expression of all these genes was found to be reduced. This effect may be the result of a direct modulation of gene expression, or secondary to growth inhibition by AHCC^®^, or even to a combination of both mechanisms. At any rate, AHCC^®^ appears to act as a signal molecule to modulate different signaling pathways resulting in lower antibiotic resistance, decreased pathogenicity and attenuated ability of *P. aeruginosa* to control the bacterial environment and to infect eukaryotic cells. Exotoxin A produced by several microorganisms, including *P. aeruginosa*, inhibits protein elongation in eukaryotic cells. Addition of AHCC^®^ to primary IEC18 cell cultures reduced the exotoxin A levels inside the cells. This effect was unrelated to modulation of TSS3/TSS6 critical genes, i.e., those encoding ExsA, Hcp and Vgr, which were actually upregulated, suggesting AHCC^®^ may act by a different mechanism such as altering the synthesis of the virulence factor. It should be noted that the secretion systems described above are important for adhesion to the cell.

To further confirm the results, we infected IEC18 cells in the presence AHCC^®^, observing that it decreased the number of bacteria inside eukaryotic cells. A number of extracellular pathogens, including *P. aeruginosa*, are able to invade epithelial cells by modulating the dynamics of host microtubules. The basic mechanism of this important strategy for improving virulence is poorly understood. Cytochalasin-induced actin alteration has been widely associated with reduction in bacterial internalization by cultured epithelial cells (Cooper, 1987. Chippendale et al., 1994. Benjamin et al., 1995). Consistent with this notion, Cyto D inhibited significantly the internalization of *P. aeruginosa* in IEC18 cells. However, the fact that this effect was greatly augmented by adding AHCC strongly suggests that the latter acts by different mechanisms.

*Pseudomonas aeruginosa* induces the production of cytokines by infected cells. This effect has been associated to the stimulation of TLR receptors, leading to activation of NFκB and MAPKs signaling pathways (Paolillo et al., 2011). We aimed to study if AHCC^®^ regulates the effect of *P. aeruginosa* on the production of cytokines by macrophage cells. As expected, bacteria induced IL6, IL10 and TNFα production and an increased phosphorylation of IκB-α and MAPKs (ERK, p38 and JNK). These stimulatory effects were prevented by AHCC^®^, with high intensity in the case of the MAPKs pathway, as even in the absence of bacteria the production of cytokines was diminished (Figure 4). From these results an indirect effect of AHCC^®^, acting on macrophage to prevent or attenuate *P. aeruginosa* infection, could be ascertained.

In conclusion, our findings indicate that AHCC^®^ may have potential as an inhibitor of *P. aeruginosa* infection, decreasing bacterial-induced virulence by modulating bacterial properties, such as growth, motility and biofilm formation, as well as decreasing the inflammatory reaction and signaling through significantly inhibiting the ERK and NFkB pathways, and bacterial internalization. Therefore, elucidation of the mechanism whereby AHCC^®^ inhibits the colonization of the intestinal epithelial cells by *P. aeruginosa* through the analysis of its adhesion and receptors may contribute to the prevention of endogenous *P. aeruginosa* septicemia arising from the intestinal tract.

## Conclusion

Our data indicate that AHCC, a nutritional supplement with prebiotic effects, acts as an anti-infective agent against *P. aeruginosa* affecting growth, motility, biofilm and virulence, resulting in a lower impact on eukaryotic cells reflected in the reduction of the secretion of inflammatory cytokines. Moreover, future preclinical and clinical investigations are needed to further validate the potential preventive and/or therapeutic efficacy of AHCC against *P. aeruginosa* infection and inflammatory conditions. We anticipate that AHCC may be employed in future therapeutic regimens to enhance the efficacy of treatment and to dampen the adverse effects of chronic treatment by antibiotics drugs currently used to alleviate immune-related and inflammatory conditions due to the *P. aeruginosa* infection.

## Data Availability Statement

The original contributions presented in the study are included in the article/Supplementary Material, further inquiries can be directed to the corresponding author/s.

## Ethics Statement

The animal study was reviewed and approved by Universidad de Granada (Ref: 277-CEEA-OH-2018).

## Author Contributions

MT-G: methodology and investigation. DC-H: validation and methodology. MM: writing—review and editing. FM: validation and writing—review and editing. OM-A: supervision, visualization, editing, and funding acquisition. AD: conceptualization, methodology, writing—reviewing and editing, and supervision. All authors contributed to the article and approved the submitted version.

## Conflict of Interest

The authors declare that the research was conducted in the absence of any commercial or financial relationships that could be construed as a potential conflict of interest.

## Publisher’s Note

All claims expressed in this article are solely those of the authors and do not necessarily represent those of their affiliated organizations, or those of the publisher, the editors and the reviewers. Any product that may be evaluated in this article, or claim that may be made by its manufacturer, is not guaranteed or endorsed by the publisher.

## Supplementary Material

Supplementary Figure 1 Formation of *P. aeruginosa* biofilm. Biofilm formation in the absence and in the presence of different concentrations of AHCC^®^ at 8 h in glass test tube and 24-well plates.

Supplementary Figure 2 Toxicity of AHCC^®^ on macrophage cells. Macrophage cell was exposed to kit reagent (LDH positive compound) and AHCC^®^ at 2 and 5 mg/of final concentration. Following incubation at 37°C for 24 h, the LDH activity was determined as described in Materials and Methods. **p* < 0.05 vs. macrophage in the presence of kit toxic compound (ANOVA followed by least significance tests).
