# Corrigendum: A standardized extract of *Lentinula edodes* cultured mycelium inhibits *Pseudomonas aeruginosa* infectivity mechanisms

**DOI:** 10.3389/fmicb.2023.1183760

**Published:** 2023-03-28

**Authors:** Mireia Tena-Garitaonaindia, Diego Ceacero-Heras, María Del Mar Maldonado Montoro, Fermín Sánchez de Medina, Olga Martínez-Augustin, Abdelali Daddaoua

**Affiliations:** ^1^Department of Biochemistry and Molecular Biology II, Pharmacy School, University of Granada, Granada, Spain; ^2^Clinical Analysis Service, Hospital Campus de la Salud, Granada, Spain; ^3^Instituto de Investigación Biosanitaria (IBS), Granada, Spain; ^4^Department of Pharmacology, School of Pharmacy, University of Granada, Granada, Spain; ^5^Centro de Investigación Biomédica en Red de Enfermedades Hepáticas y Digestivas (CIBERehd), Madrid, Spain; ^6^Institute of Nutrition and Food Technology “José Mataix”, Center of Biomedical Research, University of Granada, Granada, Spain

**Keywords:** prebiotic, AHCC^®^, *Pseudomonas aeruginosa*, motility and biofilm, secretion system and adhesion, immune response, PCR real time (qPCR), internalization

In the published article, there was an error in affiliation [5]. Instead of “[Department of Pharmacology, Pharmacy School, University of Granada, Granada, Spain]”, it should be “[Centro de Investigación Biomédica en Red de Enfermedades Hepáticas y Digestivas (CIBERehd), Madrid, Spain].”

The authors apologize for this error and state that this does not change the scientific conclusions of the article in any way. The original article has been updated.

