# Supplementary material for: A Standardized Extract of Lentinula edodes Cultured Mycelium Inhibits Pseudomonas aeruginosa Infectivity Mechanisms
Source: Front Microbiol. 2022 Mar 16;13:814448. doi: 10.3389/fmicb.2022.814448 (PMC8966770; doi:10.3389/fmicb.2022.814448)
Supplement: Supplementary file 1 [file Table_1.DOCX]

**Suppl. Table 1 Sequences of primers used in this study**.

| **Primer** | **Sequence** | **Purpose** |
| --- | --- | --- |
| **exsA.1f** | 5'**-**AGGGTAAACAAGGAAGAGGGCGTATAT-3’ | Gene which regulated system secretion type III |
| **exAs.1r** | 5'**-**CTGGACGAAGCCTTGTAGAAACTGG-3’ | Gene which regulated system secretion type III |
| **hcp.1f** | 5'**-**GTCAAGGGTGAGTCCAAGGACAAGAC**-**3’ | Gene which regulated system secretion type VI |
| **hcp.1r** | 5'**-**CATCAGGTTGGGCGTGGACTTG**-**3’ | Gene which regulated system secretion type VI |
| **vrgG.1f** | 5'**-**AGAACCAGAGCGTGCCGGAGA**-**3’ | Gene which regulated system secretion type VI |
| **vrgG.1r** | 5'**-**CCAGTAGTAGATGC CTTCCTGTTCCAT-3’ | Gene which regulated system secretion type VI |
| **16S. f** | 5'**-** TAGAGTACGGTAGAGGGTGGTGGAATTTC -3’ | Normalization of gene expression |
| **16S. R** | 5'**-** CATTGTAGCACGTGTGTAGCCCTGG -3’ | Normalization of gene expression |
| **qPCRPA0807.f** | 5'**-**CAGCGCATCTTCAACCTGGTCGCCGAG-3´ | Quantification of transcript levels by RT-qPCR |
| **qPCRPA0807.r** | 5'**-**GTCATGTCCGGGTAGCGCTGCAGGATGTTCTT-3´ | Quantification of transcript levels by RT-qPCR |
| **qPCRPA3866.f** | 5'**-**CGGAATCAGATTGCCTTGGCAGCACAGG-3´ | Quantification of transcript levels by RT-qPCR |
| **qPCRPA3866.r** | 5'**-**CCTTGACCTGGGCGGTTAGCCTGTTGACC-3´ | Quantification of transcript levels by RT-qPCR |
| **qPCRPA4370.f** | 5'**-**TTAACGCCTGGCCGCTGGACGAAGGC-3´ | Quantification of transcript levels by RT-qPCR |
| **qPCRPA4370.r** | 5'**-**CCCCAGAGGAGGAACTCGATGGCGTGGT-3´ | Quantification of transcript levels by RT-qPCR |
