# Supplementary figures and images for: A Standardized Extract of Lentinula edodes Cultured Mycelium Inhibits Pseudomonas aeruginosa Infectivity Mechanisms
Source: Front Microbiol. 2022 Mar 16;13:814448. doi: 10.3389/fmicb.2022.814448 (PMC8966770; doi:10.3389/fmicb.2022.814448)

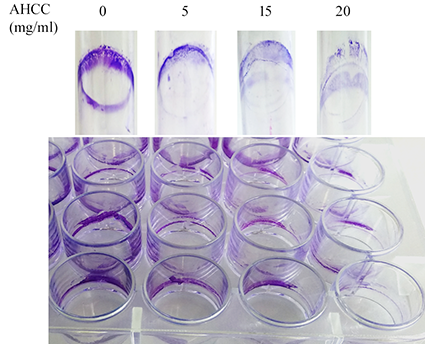

Supplement: Supplementary Figure 1 — Formation of P. aeruginosa biofilm. Biofilm formation in the absence and in the presence of different concentrations of AHCC® at 8 h in glass test tube and 24-well plates. [file Image_1.TIF]

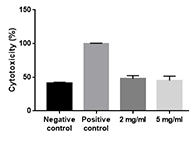

Supplement: Supplementary Figure 2 — Toxicity of AHCC® on macrophage cells. Macrophage cell was exposed to kit reagent (LDH positive compound) and AHCC® at 2 and 5 mg/of final concentration. Following incubation at 37°C for 24 h, the LDH activity was determined as described in Materials and Methods. *p < 0.05 vs. macrophage in the presence of kit toxic compound (ANOVA followed by least significance tests). [file Image_2.TIF]
